# Profiling persistent tubercule bacilli from patient sputa during therapy predicts early drug efficacy

**DOI:** 10.1186/s12916-016-0609-3

**Published:** 2016-04-07

**Authors:** Isobella Honeyborne, Timothy D. McHugh, Iitu Kuittinen, Anna Cichonska, Dimitrios Evangelopoulos, Katharina Ronacher, Paul D. van Helden, Stephen H. Gillespie, Delmiro Fernandez-Reyes, Gerhard Walzl, Juho Rousu, Philip D. Butcher, Simon J. Waddell

**Affiliations:** Centre for Clinical Microbiology, University College London, London, NW3 2PF UK; Department of Computer Science, Helsinki Institute for Information Technology HIIT, Aalto University, Espoo, Finland; Institute for Molecular Medicine Finland FIMM, University of Helsinki, Helsinki, Finland; Department of Science and Technology/National Research Foundation Centre of Excellence for Biomedical Tuberculosis Research and Medical Research Council Centre for TB Research, Division of Molecular Biology and Human Genetics, Faculty of Medicine and Health Sciences, Stellenbosch University, Western Cape, South Africa; Medical and Biological Sciences Building, University of St Andrews, North Haugh, St Andrews, Fife KY16 9TF UK; Department of Computer Science, University College London, Gower Street, London, WC1E 6BT UK; Department of Paediatrics, University College Hospital, College of Medicine of the University of Ibadan, Ibadan, Nigeria; Institute for Infection and Immunity, St George’s University of London, London, SW17 0RE UK; Brighton and Sussex Medical School, University of Sussex, Brighton, BN1 9PX UK

**Keywords:** *Mycobacterium tuberculosis*, Sputum, Transcriptional profiling, Predictive biomarker, Persistent infection

## Abstract

**Background:**

New treatment options are needed to maintain and improve therapy for tuberculosis, which caused the death of 1.5 million people in 2013 despite potential for an 86 % treatment success rate. A greater understanding of *Mycobacterium tuberculosis* (*M.tb*) bacilli that persist through drug therapy will aid drug development programs. Predictive biomarkers for treatment efficacy are also a research priority.

**Methods and Results:**

Genome-wide transcriptional profiling was used to map the mRNA signatures of *M.tb* from the sputa of 15 patients before and 3, 7 and 14 days after the start of standard regimen drug treatment. The mRNA profiles of bacilli through the first 2 weeks of therapy reflected drug activity at 3 days with transcriptional signatures at days 7 and 14 consistent with reduced *M.tb* metabolic activity similar to the profile of pre-chemotherapy bacilli. These results suggest that a pre-existing drug-tolerant *M.tb* population dominates sputum before and after early drug treatment, and that the mRNA signature at day 3 marks the killing of a drug-sensitive sub-population of bacilli. Modelling patient indices of disease severity with bacterial gene expression patterns demonstrated that both microbiological and clinical parameters were reflected in the divergent *M.tb* responses and provided evidence that factors such as bacterial load and disease pathology influence the host-pathogen interplay and the phenotypic state of bacilli. Transcriptional signatures were also defined that predicted measures of early treatment success (rate of decline in bacterial load over 3 days, TB test positivity at 2 months, and bacterial load at 2 months).

**Conclusions:**

This study defines the transcriptional signature of *M.tb* bacilli that have been expectorated in sputum after two weeks of drug therapy, characterizing the phenotypic state of bacilli that persist through treatment. We demonstrate that variability in clinical manifestations of disease are detectable in bacterial sputa signatures, and that the changing *M.tb* mRNA profiles 0–2 weeks into chemotherapy predict the efficacy of treatment 6 weeks later. These observations advocate assaying dynamic bacterial phenotypes through drug therapy as biomarkers for treatment success.

**Electronic supplementary material:**

The online version of this article (doi:10.1186/s12916-016-0609-3) contains supplementary material, which is available to authorized users.

## Background

Tuberculosis (TB) caused the death of 1.5 million people in 2013 despite potential for an 86 % treatment success rate [1]. New drug regimens are needed to maintain and improve therapy for tuberculosis, shortening treatment duration and targeting drug-resistant bacilli which complicate 3.5 % of new and 20.5 % of previously-treated TB cases [1]. The standard chemotherapy regimen for drug-sensitive TB uses combinations of 4 drugs over 6 months. The recommended treatment for multidrug-resistant TB lasts 18–24 months or more, with increasingly toxic combinations of second-line drugs. Extended periods of chemotherapy are required to remove sub-populations of *Mycobacterium tuberculosis* (*M.tb*) bacilli that persist through the early phase of antimicrobial drug treatment [2]. A recent treatment shortening trial failed to show non-inferiority despite evidence that the experimental regimens were more bactericidal in the first 4 months [3]. Evidence for the existence of persister populations recalcitrant to treatment is accumulating; quantitation of mycobacteria in serial sputum samples during treatment with regimens containing isoniazid display a characteristic biphasic pattern of killing, indicative of the presence of multiple populations of *M.tb* [4]. The exposure of sputum-derived bacilli to resuscitation-promoting factors unmasks a previously-unculturable drug-tolerant population of *M.tb* in sputum [5], and sub-populations of bacilli that only grow in liquid culture and not on solid media may represent 90 % of bacilli in sputum [6]. Similar *M.tb* populations have also been identified in chronic murine tuberculosis models [7], and generated in vitro [8]. Microfluidic systems have revealed heterogeneous mycobacterial responses to antibiotic exposure in genetically-homogenous populations, providing a basis for the generation of such sub-populations in vivo [9]. It is unlikely that the duration of tuberculosis chemotherapy will be reduced until drug regimens are identified that can kill these persister sub-populations.

Success in tuberculosis chemotherapy is measured by the proportion of patients who fail therapy or who relapse after treatment is completed; therapy is monitored by counting *M.tb* in sputum and assaying markers of drug toxicity. Clinical or microbiological parameters, such as number of lung cavities or extent of lung cavitation, *M.tb* culture positivity at 2 months [10] or bacterial load in sputum before the start of treatment [11], are associated with early treatment success but are poor predictors of treatment outcome. For example, use of a combination of biomarkers, culture negativity at 2 months and low extent of cavitation by X-ray, failed to be predictive of individuals where treatment could be shortened from 6 to 4 months [12]. However, molecular profiling assays, such as those used to identify tuberculosis disease from patient blood transcriptional signatures [13, 14], have not been applied to bacteria during patient drug therapy to assess the predictive power of dynamic bacterial responses to antimicrobial drug exposure.

The transcriptional signature of *M.tb* reflects the bacterial physiological state and offers insight into the mechanisms required to survive [15–17]. An increased understanding of which *M.tb* bacterial phenotypes survive chemotherapy during natural infection will aid the design of novel intervention strategies. To this end, *M.tb* bacilli have been profiled from in vitro models that mimic hypothesized features of *M.tb* persister populations [8, 18, 19]. Investigation of the mRNA signature of *M.tb* derived from human sputa revealed a slow/non-growing gene expression pattern alongside an accumulation of lipid bodies, a ‘fat and lazy’ phenotype [20]. In addition, by mapping the differential expression of *M.tb* respiratory pathways, the microenvironment surrounding bacilli was predicted to be, at least in part, hypoxic [20]. This observation was confirmed by 3-dimensional PET-CT imaging of human lungs that highlighted the hypoxic and dynamic nature of lesions within an individual [21]. Transcriptional profiling the response of bacilli to drug exposure in vitro has helped to define antimicrobial drug class of action and mechanisms that may influence drug efficacy [22–24]. These drug-inducible signatures, used as a bioprobe, may also identify drug-tolerant *M.tb* populations by classifying divergent responses to drug exposure [25, 26].

This study profiles sputum *M.tb* transcriptional responses during the first 14 days of standard anti-tuberculous therapy, testing the hypothesis that persister-type bacilli are the dominant population in human sputum. Drug-induced changes in *M.tb* gene expression were observed 3 days after the start of chemotherapy that were not evident at 7 or 14 days. Furthermore, the profile of bacilli derived from sputum one or two weeks after drug therapy resembled pre-treatment sputum bacilli. This suggests that bacilli with a phenotype able to survive drug therapy were already present prior to the commencement of treatment. Bacteria with a drug-responsive phenotype during days 0 to 3 were no-longer present by day 7 and were presumably killed. Importantly, we demonstrate for the first time that the diverse pathology of human disease influences the phenotype of *M.tb* bacilli in sputum. We show that microbiological (bacterial load) and clinical (number of cavities) measures of disease were predicted from the changing *M.tb* sputum signature over time, as were parameters with prognostic value (positive TTP or MBL TB test, and bacterial load at 2 months).

## Methods

### Patient sample collection, clinical and molecular parameters

Subjects with active pulmonary tuberculosis (HIV negative, sputum smear-positive pulmonary TB) were recruited in primary healthcare tuberculosis clinics in the Western Cape Province, South Africa following local ethical approval (Stellenbosch University Health Research Ethics Committee, Study no 99/039). Patients consented to be involved in the study. Details of the patients in this cohort have been reported previously [11]. Clinical parameters measuring severity of disease, such as average chest radiograph (CXR) score and number of observable cavities, were recorded. Briefly, full-sized CXRs (postero-anterior and lateral) were obtained and read by a pulmonologist blinded to patient clinical history using a standardized protocol [11]. Expectorated sputum was immediately collected into 4 M GTC solution at the clinic as previously described [27] and frozen at -80 °C. For each patient, samples were collected before the start of chemotherapy, and 3, 7 and 14 days after initiation of treatment. Standard 5 day/week clinic-based directly observed treatment (DOT) was given by routine clinic nurses using fixed-dose combinations, with dose adjustment based on patient body weight. Treatment consisted of a 2-month intensive phase of rifampicin, isoniazid, pyrazinamide, and ethambutol, followed by a 4-month continuation phase of rifampicin and isoniazid. Treatment was monitored using microbiological (BACTEC 460) time-to-positivity scores (TTP) at diagnosis (day 0), day 7 and day 14, and by the molecular bacterial load (MBL) assay [27] at diagnosis (day 0), day 3, 7, 14 and 56. Participants’ molecular, microbiological and clinical parameters are detailed in Additional file 1: Table S1.

### *M.tb* RNA extraction and amplification

Mycobacterial RNA was extracted from tuberculous sputa as previously described using the GTC/Trizol method [20]. Briefly, sputum was thawed and bacterial pellets recovered from GTC by centrifugation at 1800 g for 30 minutes. Bacterial pellets were resuspended in Trizol (Life Technologies), disrupted using a ribolyzer (MP Biomedicals) and the nucleic acid recovered in the aqueous phase after addition of chloroform. The RNA preparations were purified and DNase-treated using RNeasy columns (Qiagen). Mycobacterial RNA yield and quality were assayed using the Nano-Drop ND-1000 Spectrophotometer (NanoDrop Technologies) and Agilent 2100 Bioanalyser (Agilent Technologies). RNA samples were amplified from 100 ng total RNA using the MessageAmp II Bacteria system (Life Technologies) [16, 28]. All sputum samples were extracted and amplified together to minimize technical variation.

### Transcriptional profiling *M.tb* from sputa

Amplified *M.tb* RNA derived from 15 subjects at multiple time intervals before and during chemotherapy (totalling 52 samples) was profiled alongside *M.tb* H37Rv RNA extracted from in vitro log phase bacilli (two biological replicates hybridized in duplicate) as a standardized comparator [16, 20]. Amplified mycobacterial RNA (2 μg) was directly labelled with Cy3 fluorophore using the Universal Linkage System (ULS, Kreatech Diagnostics). Microarray hybridizations were conducted as previously described [16, 20] using an *M.tb* complex pan-genome microarray generated by the Bacterial Microarray Group at St. George’s (ArrayExpress accession number A-BUGS-41). Significantly differentially expressed genes were identified using moderated t-tests (p-value <0.05 with Benjamini and Hochberg multiple testing correction), and fold change >2 (for aerobic comparisons) and >1.5 (for sputum temporal responses) in GeneSpring 12.6 (Agilent Technologies). Samples were hierarchically clustered using Cluster and Treeview [29]. Hypergeometric probability was used to identify significantly enriched transcriptional signatures from functional classifications or from published datasets. Short time-series expression miner (STEM) was used to determine significantly represented temporal gene expression profiles (p <0.05 after Bonferroni multiple testing correction), identifying time-dependent transcriptional patterns in bacilli extracted from human sputa [30]. Significant genes identified in each comparison are detailed in Additional file 2: Table S2 and Additional file 3: Table S3.

### Quantitative RT-PCR to verify transcriptional signatures

*M.tb* cDNA (20 μL) was prepared for each sample using 1 μg RNA per reaction (Maxima 1st strand cDNA synthesis kit for RT-qPCR, Thermo Scientific) and amplified according to the manufacturer’s instructions for high GC templates. For pooled patient analyses, 10 μL first strand synthesis products were combined for each timepoint. The two *M.tb* log phase samples were amplified in triplicate and combined. Quantitative 5-plex PCR was performed on a Rotor-Gene Q platform using QuantiTect Multiplex PCR NoROX Kit (Qiagen). A total of 5 μL pooled cDNA was added per 25 μL reaction with 2× QuantiTect reaction mix and 0.2 μM primer and probes (Additional file 4: Table S4). Multiplex qPCR was run for 40 cycles according to the manufacturer’s instructions. In each 5-plex set *sigA* was used to normalize input cDNA concentration. Reactions were run in duplicate and accepted if replicate Cq values were within 1 cycle. Cq values were used to determine changes in gene expression either relative to day 0 (before treatment) or to log phase bacilli using the 2-(delta delta Cq) method [31].

### Modelling associations between RNA signatures and microbiological/clinical parameters

Gene expression data were gathered into a matrix *X*^*q*^ ∈ ℝ^52 × 4456^ for quantile normalized and *X*^*d*^ ∈ ℝ^37 × 4456^ for day 0 normalized data, with samples (patient, timepoint) in rows and genes in columns. Both matrices were standardized so each column had a mean of 0 and standard deviation of 1. Unsupervised dimensionality reduction technique principal component analysis (PCA) [32] was applied to the gene expression matrix *X*^*q*^ to visualize temporal movement of the samples in principal component space. To investigate whether the gene expression trajectories over the first two weeks of therapy followed classifiable patterns, the patients (k) were divided into two classes based on the variance of the PC data points along the principal component axis: *y*_*k*_ = − 1, *if var*(*PC*1) > *var*(*PC*2) *and y*_*k*_ = 1, *otherwise*. That is, the class corresponds to the principal component axis along which the PC data points of a patient has larger variance. These target classes were modelled using support vector machine (SVM) [33] with gene expression data or clinical/microbiological variables as the predictors to define gene signatures or clinical variables, after applying stability selection or forward feature selection, respectively, that explained the patient trajectories.

The relationships between the *M.tb* transcriptional profile and patient clinical/microbiological variables were defined using machine learning methods. Time-to-positivity was modelled with *X*^*q*^ and other clinical variables with *X*^*d*^*.* Firstly, stability selection [34], implemented according to the SCoRS framework, was used for feature selection [35]. A total of 500 sub-sampling iterations were performed with a sub-sample that consisted of 500 features (columns in *X*) and 2/3 of the samples (rows in *X*). Stability selection yielded a set of stable, most predictive features of a particular clinical variable, which was used to train a regression or classification model. To optimize the number of features and to determine the predictive performance of the models, nested leave-one-out cross-validation was carried out, in which the inner loop determined the optimal number of variables and the outer loop the error of the model (Additional file 5: Figure S1). The reported errors are the average prediction errors of the test samples in the outer loop. Continuous clinical variables time-to-positivity and average chest X-ray score were modelled with L1-norm regression (Lasso) [36], and the performance evaluated with root mean squared error (RMSE) and Pearson correlation coefficient between the fitted and true values. Binary clinical variables, TB test positivity at week 8 (MBL or TTP positive test vs*.* negative tests at week 8)*,* bacterial load at day 0 (MBL >= 10^6^ bacilli defined as high, MBL<10^6^ classed as low)*,* and rate of decline in bacterial load from day 0 to 3 (ratio of MBL at day 3 relative to day 0, MBL ratio >= 10 defined as high, MBL ratio <10 classed as low) were modelled with SVM. Molecular bacterial load at week 8 was modelled with binary SVM by dividing the response values into two bins based on the distribution of the values (MBL week 8 <300 defined as low, MBL week 8 >=300 classed as high). Performance of the SVM models was measured with average classification error, the fraction of misclassified test samples in cross-validation. All analyses, except the SCoRS algorithm, were implemented with Matlab and R using their built-in functions.

## Results

The transcriptional states of *M.tb* bacilli derived from human sputa were mapped through the early stages of chemotherapy. *M.tb* bacilli were isolated from the sputa of 15 patients before chemotherapy was begun and then at 3, 7 and 14 days of therapy with the standard regimen (isoniazid, ethambutol, rifampicin and pyrazinamide). All patients had non recurrent active tuberculosis and were treated successfully for drug-sensitive tuberculosis and all patients were culture negative at 3 months.

### The ‘fat and lazy’ transcriptome of pre-chemotherapy sputum-derived bacilli

**Fig. 1:**
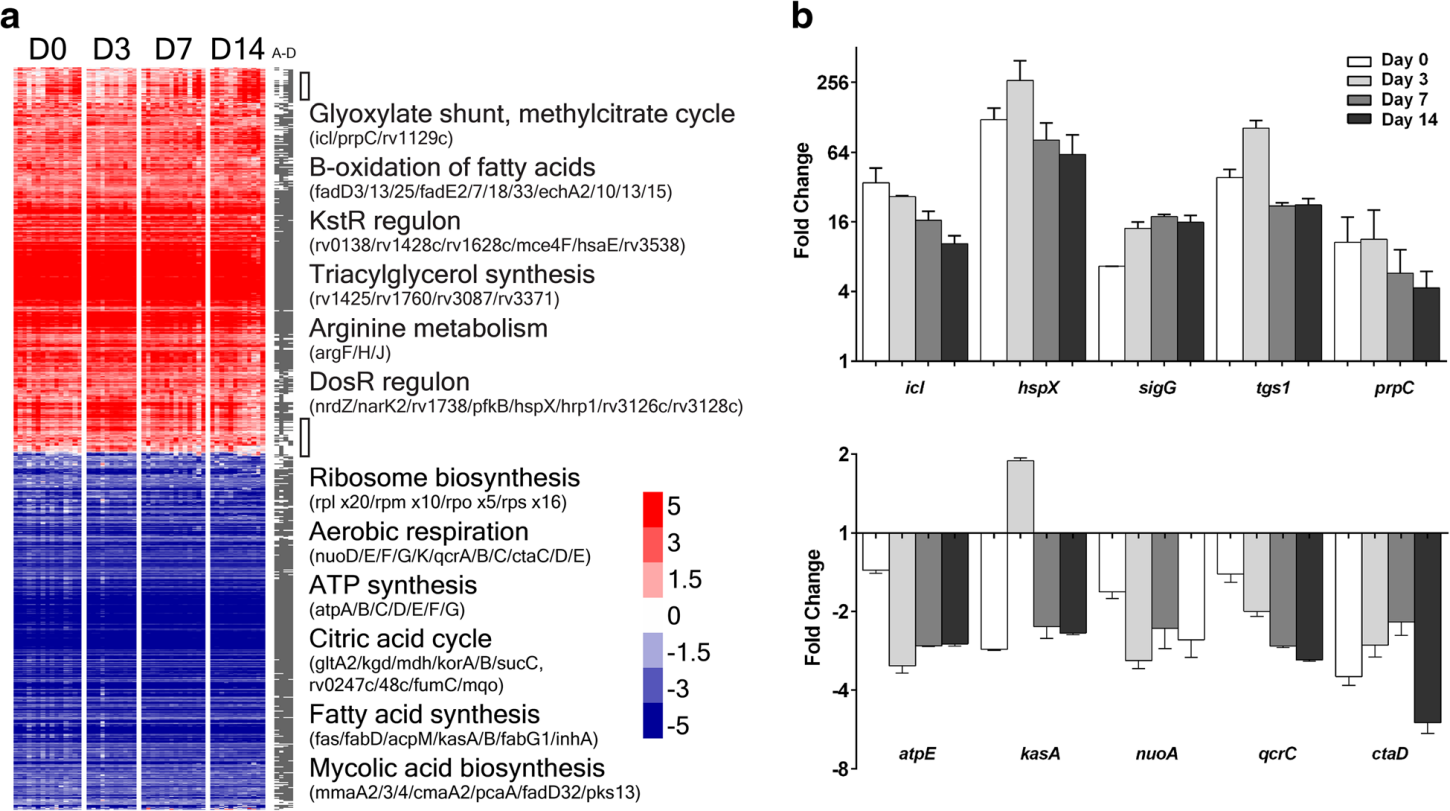
The transcriptional signature of *M.tb* bacilli in sputa relative to aerobic log phase growth before chemotherapy (D0) and 3 days (D3), 7 days (D7) and 14 days (D14) after beginning standard regimen drug therapy. **a** The 1337 genes significantly differentially expressed at any sputum timepoint compared to axenic log phase bacilli are displayed as rows; each patient/timepoint as columns. Colouring details fold change relative to log phase bacilli; red denoting up-regulation, blue repression. Adaptations to mycobacterial respiratory and metabolic state are summarized as text, listing key indicator genes that were significantly regulated at D0. *Grey columns* (A-D) mark in which comparison each gene was identified. *Clear boxes* signpost clusters of genes that were differentially expressed over time. **b** Quantitative RT-PCR verification of 10 genes as key indicators of *M.tb* physiological state measured in patient samples before treatment (day 0) and after 3, 7 and 14 days drug therapy. Genes induced in sputum *icl*, *hspX*, *sigG*, *tgs1* and *prpC* and genes repressed in sputum *atpE, kasA, nuoA*, *qcrC* and *ctaD*. Log2 expression ratios are plotted; the y-axis detailing fold change relative to log phase bacilli. Error bars mark the standard error of the mean

The transcriptional profile of *M.tb* bacilli from sputa before the start of chemotherapy (day 0) was defined relative to bacilli grown in vitro in axenic log phase culture. A total of 1083 genes were significantly differentially expressed in sputum-derived bacilli (day 0) compared to log phase growth, which revealed adaptations to respiratory and metabolic pathways (Fig. 1a/b, Additional file 2: Table S2, Additional file 5: Figure S2 and Figure S3). The sputum transcriptional signature was dominated by the repression of genes involved in intermediate metabolism (II.A) and ribosome synthesis (II.A.1). These functional categories (defined by Cole et al. [37]) were significantly over-represented amongst down-regulated genes (hypergeometric probabilities of 9.4x10^-15^ and 3.1x10^-30^, respectively). This drop in markers of cellular activity was accompanied with the repression of central metabolism and lipid biosynthesis, including the citric acid cycle (*gltA2*, *kgd*, *mdh*, *korA/B*, *sucC*, *rv0247c/48c*, f*umC* and *mqo*), FAS-1 (*fas*), FAS-II (*fabD*, *acpM*, *kasA*/*B*, *fabG1* and *inhA*), and mycolic acid synthesis and modification (*mmaA2*/*3*/*4*, *cmaA2*, *pcaA*, *fadD32* and *pks13*) pathways [38, 39]. Conversely, genes involved in the glyoxylate shunt pathway and methylcitrate cycle (*icl*, *prpC* and *rv1129c*), catabolism of cholesterol and fatty acids [40], and the predicted triacylglycerol synthases (*rv1425*, *rv1760*, *rv3087* and *rv3371*) [41] were induced in sputum-derived bacilli. Thus, the *M.tb* sputum transcriptome suggests that central carbon metabolism and general metabolic activity is reduced in bacilli expectorated from the lungs of tuberculosis patients, with an increased emphasis on lipolytic pathways. The transcriptomic evidence also predicted that the respiratory state of bacilli was altered in sputum with the down-regulation of NADH dehydrogenase I (*nuoD*/*E*/*F*/*G*/*K*), cytochrome C reductase (*qcrA*/*B*/*C*) and aa3 cytochrome C reductase (*ctaC*/*D*/*E*) pathways that are utilized in aerobic and microaerophilic conditions [42]. Correspondingly, genes involved in nitrate reduction (*narK2*/*3*) were induced, indicating the potential for a switch to alternative electron acceptors and anaerobic respiration. These adaptations are likely to be mediated in part by the transcriptional regulator DosR (DevR) that is induced by hypoxia and nitric oxide [43], with several DosR-regulated genes significantly up-regulated in sputa (*nrdZ*, *narK2*, *rv1738*, *pfkB*, *hspX*, *hrp1*, *rv3126c* and *rv3128c*). Energy metabolism was also affected as ATP synthase genes (*atpA*-*G*) were repressed in sputum-derived bacilli; unsurprisingly the functional categories for aerobic respiration (1.B.6.a) and ATP-proton motive force (1.B.8) [37] were significantly enriched in those genes down-regulated in sputum (hg p-values 6.5x10^-5^ and 3.2x10^-8^, respectively). The differential expression of these pathways and gene families are summarized in Fig. 1a and Additional file 5: Figure S3. In addition, mRNA profiles of 10 metabolic and respiratory indicator genes (*icl*, *hspX, sigG*, *tgs1, prpC*, *atpE*, *kasA, nuoA*, *qcrC* and *ctaD*) were confirmed by quantitative RT-PCR (Fig. 1b), the high concordance of expression ratios between contrasting assays validating our approach.

### The *M.tb* mRNA signature 7 and 14 days into chemotherapy resembles untreated bacilli

**Fig. 2:**
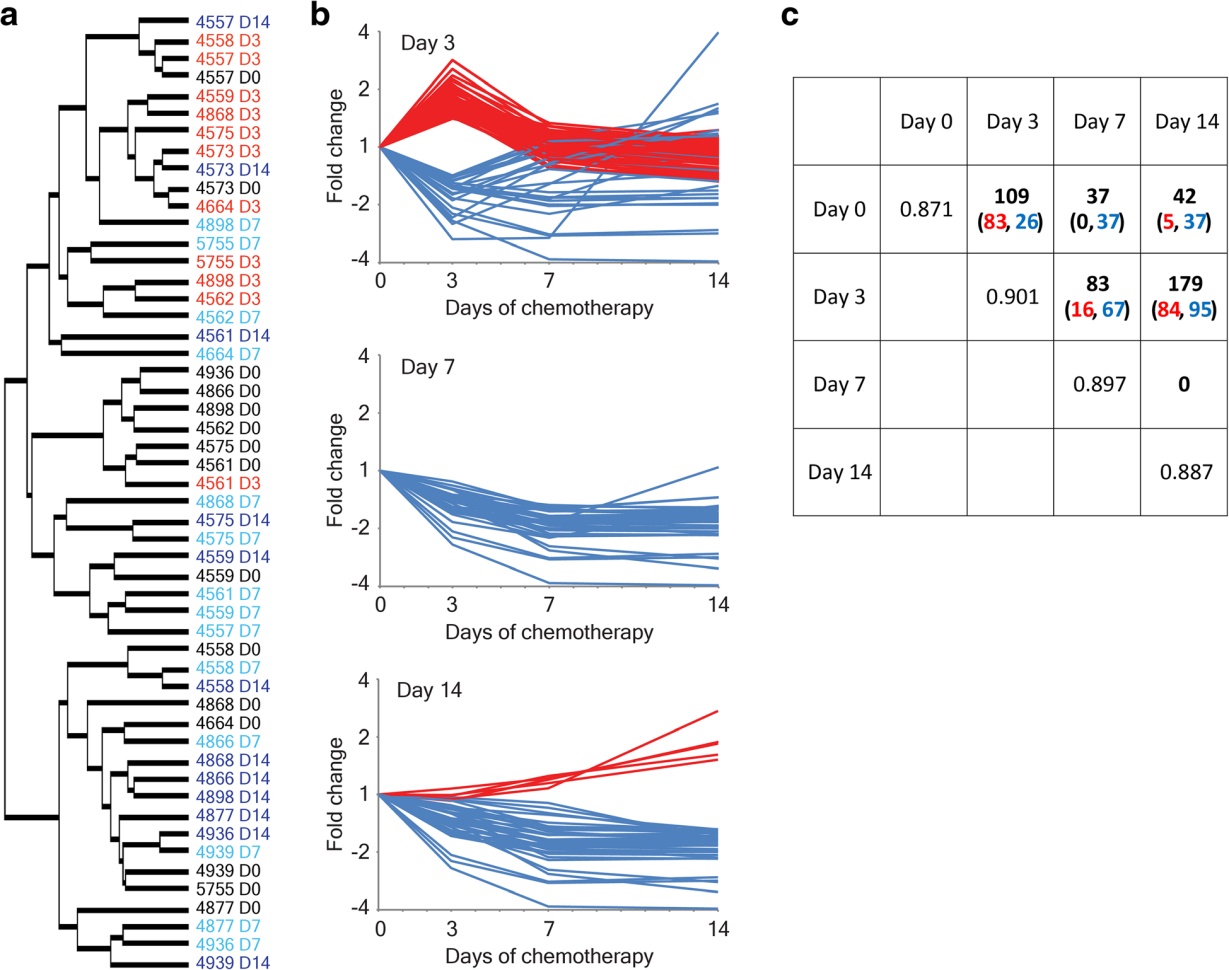
The *M.tb* mRNA signatures 7 and 14 days into chemotherapy resemble untreated bacilli. **a** Hierarchical clustering of *M.tb* transcriptional profiles derived from sputa before the start of drug therapy (D0, black) and 3, 7 and 14 days into chemotherapy (D3, red; D7, blue; D14, purple). The dendrogram is derived from clustering all genes (4456) and all sputum samples (52) after median centring. Individual patient study numbers are marked. **b** *M.tb* genes significantly differentially expressed over time in sputa; at day 3 (*top panel*), day 7 (*middle panel*) and day 14 *(bottom panel*) compared to pre-chemotherapy (day 0) bacilli. Log2 expression ratios are plotted 3, 7 and 14 days after the start of drug therapy; the y-axis detailing fold change relative to day 0. Red colouring marks up-regulation, blue repression. **c** Table detailing *M.tb* responses to the early stages of drug therapy. The number of genes significantly induced (red) or repressed (blue) in pairwise comparisons of sputum time points are marked in the matrix. The mean Spearman’s rank correlation scores between samples at each time interval are also detailed (across the *diagonal*), demonstrating that variation in replicate sputa at each time point did not bias the statistical testing

We were able to successfully extract, amplify and profile mycobacterial RNA from sputa, after 3, 7 and 14 days of standard chemotherapy despite falling bacterial viable counts. Hierarchical clustering revealed that the *M.tb* signatures from sputum-derived bacilli 14 days after the start of drug therapy were most similar to pre-chemotherapy (day 0) bacilli and day 7 bacilli, and that profiles from day 3 clustered away from the other time intervals (Fig. 2a, Additional file 5: Figure S4). These observations from unsupervised hierarchical clustering were supported by the results of pairwise significance testing. A total of 109 genes were identified as significantly differentially expressed at day 3 compared to day 0; in comparison to 37 or 42 genes that were significantly different between day 0 and days 7 or 14, respectively (Fig. 2b/c, Additional file 3: Table S3). No genes were significantly divergently expressed between day 7 and day 14 time intervals. The variation between sample profiles at each time point were similar (Spearman’s rank correlations of 0.871 to 0.901, Fig. 2c), suggesting that the number of statistically significant genes identified in each comparison could be used reliably as a measure of similarity. A pattern emerged, analogous to the unsupervised hierarchical clustering, demonstrating that *M.tb* responses at 7 and 14 days during chemotherapy were most similar to that of bacilli before drug therapy had begun.

The transcriptional signature from *M.tb* bacilli that have persisted in patients through 14 days of standard drug therapy was similar to the pre-chemotherapy profile; for example, 501 of the 528 genes induced in sputum at day 0 relative to log phase bacilli were also up-regulated in sputum at day 14 (Fig. 1a, Additional file 2: Table S2). Thus, the gross changes to metabolic and respiratory pathways are comparable between sputum-derived bacilli before and during chemotherapy. However, the magnitude of transcriptional adaptations was elevated at day 14 compared to day 0; using aerobic log phase bacilli as a comparator, 594 genes were significantly induced (605 genes repressed) at 14 days in contrast to 528 up-regulated genes (555 down-regulated genes) at day 0 (Additional file 5: Figure S2, Additional file 2: Table S2). A direct comparison of day 7 or day 14 transcriptional profiles to day 0 revealed that *M.tb* gene expression was predominately repressed with time (Fig. 2b/c); 37 genes were significantly down-regulated at day 7 compared to day 0, and 37 genes were repressed at day 14 (29 overlapping with day 7 signature), with five genes induced (Fig. 2b/c, Additional file 3: Table S3). Of the genes that were down-regulated over time, 88 % were also repressed in sputum relative to log phase bacilli, suggesting that the non/slow-growing phenotype of sputum-derived bacilli was further enhanced over time and through chemotherapy. These genes encode ribosomal proteins (*rpmH* and *rpsJ*), ribonucleotide reductase subunits (*nrdH* and *nrdI*) involved in the generation of precursors for DNA synthesis, and type VII secretion system elements (*esxA*, *esxB*, *esxH*, *esxN*, *esxO*, *esxV*, *espD* and *espG*3). In contrast, *prpC*, a methylcitrate synthase up-regulated in day 0 bacilli relative to log phase bacilli was down-regulated with drug therapy, indicating that specific modifications to metabolic pathways may occur over time.

### Evidence of anti-mycobacterial drug action 3 days into treatment

**Fig. 3:**
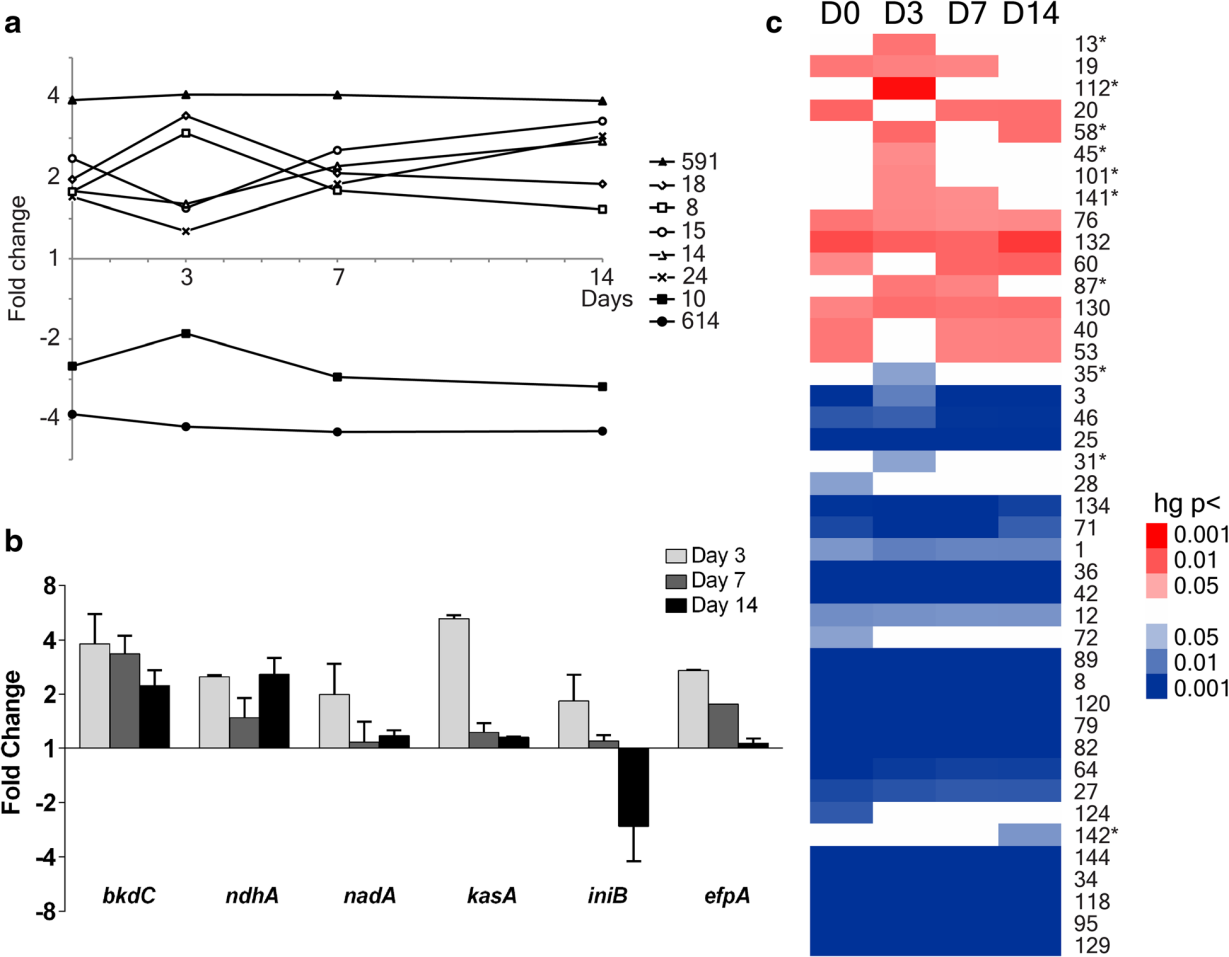
The changing *M.tb* transcriptional pattern in sputum over time, highlighting day 3 as an inflection point. **a** Significantly represented temporal gene expression profiles in *M.tb* bacilli extracted from sputa relative to log phase bacilli using short time-series expression miner (STEM). The numbers of genes assigned to each gene expression curve are marked. **b** Quantitative RT-PCR defining day 3-specific induction of *bkdC*, *ndhA*, *nadA*, *kasB*, *iniB* and *efpA* plotted at 3, 7 and 14 days after the start of drug therapy. Log2 expression ratios are plotted; the y-axis detailing fold change relative to day 0 sputum bacilli. Error *bars* mark the standard error of the mean. **c** Significantly enriched gene clusters, previously defined in response to antimicrobial drugs [23], in *M.tb* derived from sputum at 0, 3, 7 and 14 days after the start of chemotherapy. Greater statistical significance is indicated by increasing depth of colour (minimum hypergeometric p-value <0.05). Gene clusters in red overlap with genes up-regulated (blue, down-regulated) in sputum compared to aerobic log phase bacilli. Gene clusters are labelled numerically as in [23]. Ten gene clusters were identified as significantly enriched only after drug treatment had started (marked with asterisks). Gene clusters 101, 31, 35 and 142 reflect exposure to pyrazinamide and rifampicin; cluster 45 to rifampicin alone; and cluster 87 to pyrazinamide alone. Of the ten enriched drug responsive gene clusters, six were only significant at day 3

An inflection point at day 3 of chemotherapy was identified by mapping significantly represented temporal gene expression profiles (Fig. 3a). Of the eight significantly represented gene curves describing the *M.tb* response to early drug therapy, six were modified at day 3 in comparison to day 0, 7 or 14 time intervals. The inference from this observation, that the mRNA signature 3 days after the start of chemotherapy was distinct from the other sputa time points, was reinforced by hierarchical clustering (Fig. 2a, Additional file 5: Figure S4) and significance testing (Fig. 2b/c). A total of 83 genes were significantly up-regulated 3 days after the start of drug treatment compared to pre-chemotherapy bacilli. The majority of these genes were also induced at day 3 relative to both 7 and 14 days of drug therapy (Additional file 5: Figure S5) suggesting that the day 3 transcriptional pattern represented a short-lived response to the start of drug treatment. This mRNA signature consisted of a diverse subset of genes involved in intermediary metabolism (*argF*, *bkdC*, *galU*, *hisA*, *hisF*, *icl1*, *moaD1*, *pfkB*, *phoT* and *pstC1*), cell wall metabolism (*alr*, *fbpD* and *murC*) and response to oxidative stress (*alkB*, *cyp136* and *rv0547c*). The alternative sigma factor *sigG*, induced as part of the RecA-independent DNA damage response and implicated in the regulation of detoxification systems [44], was also up-regulated. Perhaps importantly, three genes involved in the export of antimicrobial drugs were induced at day 3: 1) *rv1218c*, encoding a putative ATP-dependent efflux pump regulated by RaaS (rv1219c) that is functionally significant in the response to antimicrobial drugs in non-permissive growth conditions [45]; 2) *rv2688c*, a predicted ABC fluoroquinolone efflux pump [46]; and 3) r*v3066*, a mycobacterial transcriptional regulator of the multidrug efflux pump Mmr (Rv3065) that has been shown to influence mycobacterial resistance to multiple toxic compounds [47]. Quantitative RT-PCR confirmed the induction of *bkdC*, involved in branched amino acid metabolism, *ndhA,* encoding a nonproton-pumping type II NADH dehydrogenase, and *nadA,* a quinolinate synthetase involved in NAD biosynthesis, at day 3 relative to day 0 (Fig. 3b). The maximal induction of *sigG*, *hspX* and *tgs1* at day 3 was also verified by qRT-PCR (Fig. 1b).

To further explore the relationship between sputum mRNA signatures and drug action in vivo, the transcriptional adaptations to a range of antimicrobial drugs in vitro [23] were mapped to the *M.tb* sputa profiles (Fig. 3c). Ten gene clusters (responsive to drug exposure) were identified to overlap with sputum transcriptional signatures exclusively after drug treatment had started. Transcriptional patterns reflecting exposure of bacilli to pyrazinamide and rifampicin were observed providing molecular evidence of in vivo drug action. Of the ten enriched drug responsive gene clusters, six were only significant at day 3. Quantitative RT-PCR of genes that are highly up-regulated after *M.tb* exposure to cell-wall inhibitors [22, 23], exemplified by *iniB* and *efpA*, were specifically induced at day 3 compared to drug-free day 0 bacilli (Fig. 3b). The expression of these benchmark genes for the activity of isoniazid and ethambutol decreased at 7 and 14 days relative to day 3. Thus, the transcriptional signature of bacilli 3 days into chemotherapy may reflect in vivo drug-induced changes; however, many of these responses to drug action were short-lived and were not evident 7 or 14 days after the start of drug therapy. We hypothesize that these signatures represent the killing of a drug-sensitive *M.tb* population after 3 days chemotherapy, and reveal the presence of a pre-existing drug-tolerant *M.tb* population that persists through early drug treatment.

### *M.tb* transcriptional signatures reflect patient disease and predict treatment progress

**Fig. 4:**
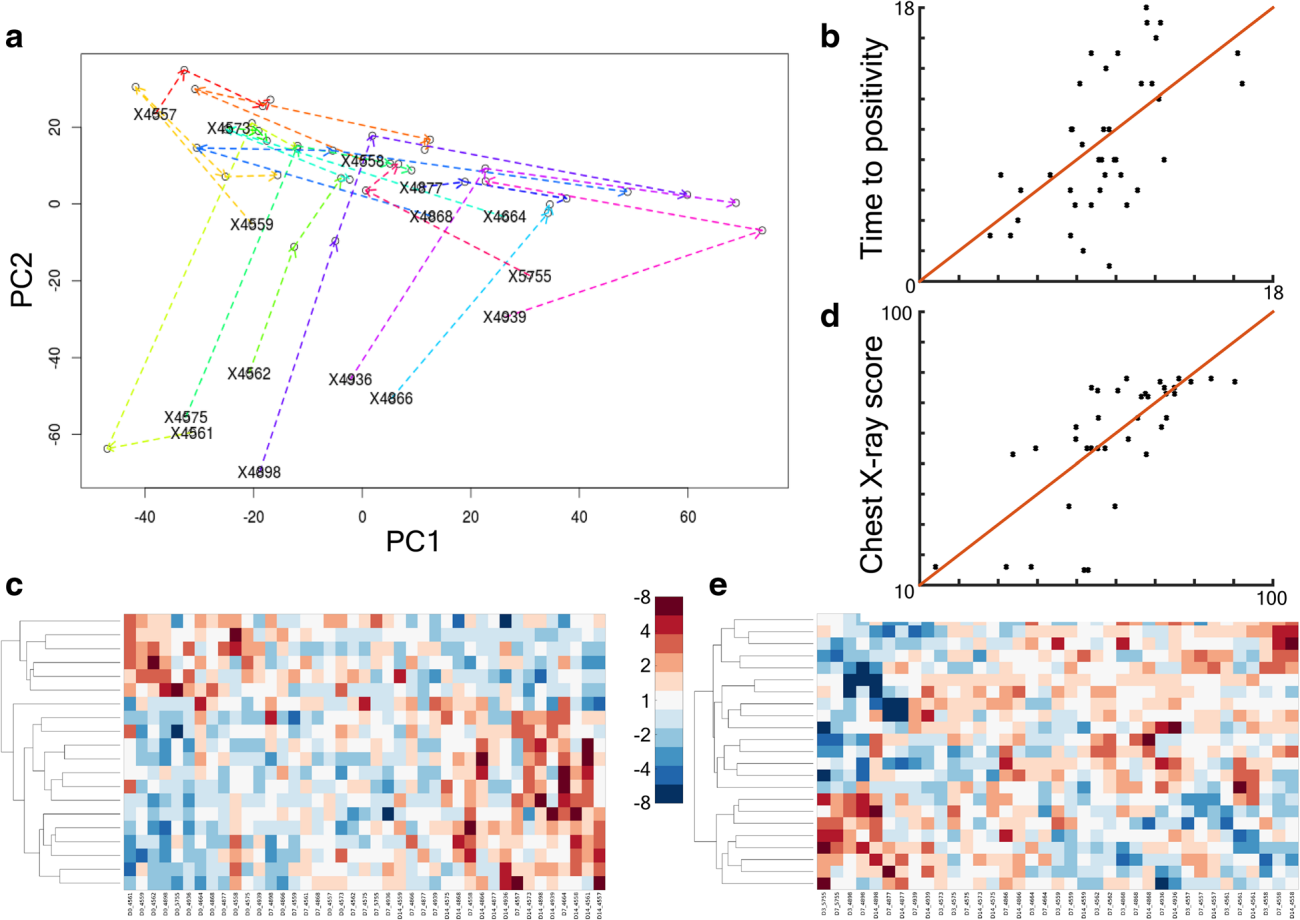
The associations between patient clinical and microbiological parameters and *M.tb* sputa transcriptional signatures. **a** *M.tb* responses to drug therapy result in contrasting patient trajectories as defined by principle component analysis (PCA). The first (PC1) and second (PC2) principle components of the *M.tb* transcriptional signatures from each patient at day 0, 3, 7 and 14 are plotted. Each point represents an *M.tb* mRNA profile derived from a patient (coloured individually), *arrows* and *dashed* lines mark the direction and distance of movement of each patient from day 0 to day 14. Patient study identifiers are plotted at day 0. **b**/**c** Fitted (x-axis) against true (y-axis) time-to-positivity (TTP) values of test samples modelled using the displayed set of 20 genes. y=x *red line* indicates ideal performance of the model; RMSE 3.6 hours, Pearson correlation coefficient 0.59, p-value of 4.4×10^-5^. **d**/**e** Fitted (x-axis) against true (y-axis) chest x-ray scores of test samples modelled using the displayed set of 23 genes. y=x *red line* indicates ideal performance of the model; RMSE 13.9 CXR score, Pearson correlation coefficient 0.73, p-value 3.7×10^-7^. *RMSE* root mean squared error, *CXR* chest radiograph

All patients in this cohort were treated successfully for tuberculosis: nine of 15 patients were culture negative at 2 months, and all patients were culture negative at 3 months. Microbiological time-to-positivity scores (TTP) and molecular bacterial load (MBL) estimates were recorded alongside clinical parameters measuring severity of disease (chest radiograph score and the number of observable cavities) (Additional file 1: Table S1). Unsupervised principal component analysis (PCA) plotting the first and second principle components of the *M.tb* transcriptional signatures from each patient over the first 2 weeks of treatment time showed that *M.tb* responses followed distinct trajectories (Fig. 4a). This suggested that *M.tb* responses to drug therapy may differ between patients and that these bacterial mRNA signatures may reflect patient disease severity or predict treatment progress. Interestingly, two directions of travel emerged at 0–3 days with South-North and East-West trajectories most common (Fig. 4a and Additional file 5: Figure S6). Therefore, the PC trajectories were grouped into two classes according to the direction of movement in principal component space (South-North or East-West). Unsurprisingly, since the PCA patterns are a representation of the transcriptional responses, support vector machine classification (SVM) confirmed that PC trajectory class could be predicted from the gene signatures (classification error of 8 %, linear kernel). Furthermore, SVM was performed to test whether clinical or microbiological parameters collected for each patient would affect *M.tb* profiles such that the patient’s membership in the South-North or East-West class could be forecasted. The number of observed cavities in the lung (at diagnosis) and the bacterial load at 8 weeks (MBL measurement) predicted PC trajectory class with a classification error of 13 %, a success rate of 87 %. These observations demonstrated that the expression profile of *M.tb* in sputum was associated with specific measurable patient parameters, that variability in clinical and microbiological manifestations of disease could be detected in the bacterial sputa signatures, which may also be of prognostic value.

These findings were expanded using L1-norm regression to model the impact of continuous clinical and microbiological variables on *M.tb* sputa transcriptional profiles. Gene signatures were characterized that optimally designated time-to-positivity (day 0, day 7, day 14) with a RMSE (root mean squared error) of 3.6 hours. The fitted compared to true data are plotted in Fig. 4b/c, alongside the predictor gene set. The Pearson correlation coefficient between true and predicted values was 0.59 with p-value 4.4x10^-5^. Transcriptional patterns were also identified that correctly reflected patient chest X-ray score (at day 0) with a RMSE of 13.9 CXR score, Pearson correlation coefficient between true and predicted values of 0.73 with p-value 3.7x10^-7^ (Fig. 4d/e). *M.tb* expression profiles were also defined that were able to discriminate between high (>=10^6^ bacilli measured by MBL) and low (<10^6^ MBL bacilli) bacterial load at day 0 using a linear SVM (with classification error of 5 %). Thus, these clinical and microbiological parameters representing severity of disease and number of bacteria correlated with measurable changes in the *M.tb* transcriptional profile in sputa over the first 14 days of drug therapy. Notably, *M.tb* sputa signatures also predicted measures of early treatment response. The rate of decline in bacterial load from day 0 to day 3 (measured by MBL) was forecast with a Gaussian SVM model with a classification error of 3 %. TB test positivity at week 8 (positive wk 8 TTP or MBL assay) was correctly classified using a Gaussian SVM with a classification error of 11 % (successfully calling treatment progress 89 % of the time). Similarly, a high or low molecular bacterial load at week 8 could be determined from the sputa transcriptional dataset using SVM with a classification error of 0 %, 100 % test accuracy. Thus, the changing transcriptional profile of *M.tb* derived from human sputa reflects features relevant to patient disease and may be used to predict early treatment success.

## Discussion

*M. tuberculosis* bacilli derived from human sputa 2 weeks after the start of treatment should be portrayed as ‘fat, lazy and indifferent’ to drug therapy. In this study we were able to use genome-wide transcriptional profiling to map the mRNA signatures of *M.tb* from the sputa of clinically well-defined patients through the first 2 weeks of treatment, offering insight into the phenotypic state of bacilli that persist through chemotherapy. This contributes to our understanding of the in vivo efficacy of combination drug regimens and may aid novel anti-mycobacterial drug development programs targeting drug-persistent bacilli. The pre-chemotherapy *M.tb* signature defined here extends and validates the findings of a previous microarray study [20], confirming the *M.tb* sputa profile in a larger cohort of clinically-defined patients collected independently in a different country and assayed using a complementary profiling methodology. In addition, single gene inferences were verified by qRT-PCR using a panel of benchmark indicator genes.

The *M.tb* mRNA signatures 7 and 14 days after the start of drug therapy were most similar to that of bacilli before drug therapy had begun, as evidenced by hierarchical clustering and differential gene expression analysis. Transcriptional changes at 7 and 14 days also indicated that the *M.tb* day 0 sputum phenotype was enhanced with chemotherapy, with transcriptional markers of an active metabolic state further repressed over time. This mirrors findings from a recent study of *M.tb* in sputa using multiplex qRT-PCR that showed transition to a slow-growing low-metabolic activity phenotype after the start of drug treatment [48]. The broad changes in *M.tb* sputum physiology reflected in these transcriptional patterns were conserved between studies, where day 14 signatures were more similar to day 7 than day 2 [48] or day 3 (this study). However, we argue here that by using a well-defined log phase *M.tb* population as a comparator (as applied in many studies) enabled the pre-chemotherapy (day 0) *M.tb* population to be characterized [20], allowing us to propose that a pre-existing, slow/non-replicating and likely drug-tolerant *M.tb* population dominates sputum before, and after, early drug treatment. This conclusion is supported by evidence from other studies identifying large non-culturable on solid media or rpf-dependent *M.tb* sub-populations in sputum prior to treatment [5, 6].

An inflection point in the mRNA profile 3 days after the start of drug treatment, of stress-responsive genes including mediators of drug efflux, likely represents the effects of anti-TB drug action, using bacterial mRNA responses as a bioprobe for cidal drug action during natural disease. This transcriptional signature was only observed 3 days into therapy and was absent after 7 or 14 days, where the mRNA patterns were more similar to sputa bacilli before treatment. We hypothesize that the mRNA signature at day 3 marks the killing of a drug-sensitive sub-population of bacilli, since these responses are not detected after drug exposure in phenotypically drug-tolerant bacilli [26]. By mapping previously-characterized in vitro *M.tb* transcriptional responses to antimicrobial drugs, we were able to define the in vivo action of standard regimen chemotherapy in a clinical setting. The expression of isoniazid-inducible benchmark genes (*iniB*, *efpA*) at day 3 alone suggest that the sputum bacterial population profiled here 1 week after the start of chemotherapy no longer respond to the antimicrobial actions of isoniazid. This finding supports the Walter et al. study that detected an isoniazid response in sputa bacilli 2 days after the start of drug therapy that disappeared 7 days into treatment [48], and mirrors evidence describing the bimodal early bactericidal activity of isoniazid in combination regimens [2, 4].

This study should be seen as capturing changes in *M.tb* mRNA abundance rather than differential transcriptional regulation since the structure of the underlying *M.tb* population is unknown. Therefore, the transcriptional read-out described here represents the average changes in gene expression from shifting mycobacterial sub-populations found in sputum. As such, lack of a response to drug therapy might reflect limited exposure of bacilli to antimicrobial drugs [49] and/or the presence of drug-tolerant bacterial populations that are able to survive drug treatment. The development of mycobacterial phenotypic drug tolerance has been described in many in vitro models for tuberculosis [8, 18, 19] and is often associated with a slow/non-replicating bacterial state, such as that inferred from the sputum *M.tb* mRNA signature. Moreover, recent observations using single cell reporter technology described the development of non-growing but metabolically-active mycobacterial sub-populations during murine infection [50]. Our study further emphasizes the significance of drug-tolerant bacilli in human tuberculosis, identifying a transcriptionally-active *M.tb* population that persists through 2 weeks of standard chemotherapy.

Modelling *M.tb* gene expression in the sputa of patients over time revealed that the transcriptional pattern of bacilli varied from patient to patient with drug treatment. Correlation of patient indices of disease severity with bacterial mRNA signatures showed that basic microbiological (time-to-positivity, bacterial load at day 0) and clinical (chest radiograph score) parameters were reflected in the divergent *M.tb* responses. These associations suggest that factors such as bacterial load and disease pathology influence the host-pathogen interplay and, thus, the phenotypic state of bacilli, which in turn might affect the natural history of patient disease. Importantly, this demonstrates for the first time that the diverse pathology of tuberculosis affects measurable changes on the phenotype of *M.tb* bacilli in sputa. Notably, transcriptional signatures were also identified that predicted measures of early treatment success (rate of decline in bacterial load over 3 days, MBL or TTP positivity at 2 months, bacterial load at 2 months). Although this study was not significantly powered to test these signatures, these observations highlight the potential use of assaying dynamic bacterial phenotypes through drug therapy as biomarkers for treatment efficacy. These data are supported by recent evidence suggesting that higher percentages of lipid-body-positive acid-fast bacilli 3–4 weeks after the start of treatment, rather than initial baseline counts, correlated with treatment failure or relapse [51]. We demonstrate here a novel concept and proof-of-principle that proposes using the changing transcriptional state of infecting bacilli to monitor treatment success.

## Conclusions

This study defines the transcriptional signature of bacilli in sputum after 2 weeks of drug treatment, mapping the molecular phenotype of persister-type bacilli. We demonstrate for the first time that variability in clinical manifestations of disease are detectable in bacterial sputa signatures, and that the changing *M.tb* mRNA profiles 0–2 weeks into chemotherapy predict the efficacy of treatment 6 weeks later. These findings advocate a novel biomarker discovery strategy, profiling the phenotype of infecting bacteria, to find predictive markers of treatment success that are desperately needed in clinical trials and to stratify at-risk patients in the clinic.

### Availability of data and materials

Fully annotated microarray data have been deposited in ArrayExpress, accession number: E-MTAB-3872.

## Additional files

Additional file 1: Table S1. Participants’ molecular, microbiological and clinical parameters. (XLSX 37 kb) Additional file 2: Table S2.
*M.tuberculosis* genes significantly differentially expressed in bacilli derived from sputum before the start of chemotherapy (day 0) and 3, 7 and 14 days during treatment compared to log phase aerobic growth. (XLSX 352 kb) Additional file 3: Table S3.
*M.tuberculosis* genes significantly differentially expressed in sputa over time. (XLSX 48 kb) Additional file 4: Table S4. Quantitative RT-PCR multiplex primer and probe sequences. (XLSX 11 kb) Additional file 5:
**Figure S1**. An illustration of the computation process to test associations between transcriptional signatures and patient variables. **Figure S2**. Venn diagrams describing the overlapping transcriptional signatures of bacilli in sputum relative to aerobic log phase growth. **Figure S3**. Box and whisker plots mapping the differential expression of gene families. **Figure S4**. Hierarchical clustering of the mean *M.tb* transcriptional profiles derived from sputa. **Figure S5**. Venn diagram highlighting genes significantly induced 3 days after the start of drug therapy. **Figure S6**. Contrasting patient trajectories as defined by principle component analysis plotting day 0 and day 3 timepoints only. (PDF 526 kb)

## Abbreviations

hg p-values: Hypergeometric probabilities
*M.tb*: *Mycobacterium tuberculosis*
MBL: Molecular bacterial load
PCA: Principal component analysis
RMSE: Root mean squared error
SVM: Support vector machine
TB: Tuberculosis
TTP: Time-to-positivity

## References

[1] World Health Organisation. Global tuberculosis report. Switzerland: WHO Press; 2014.

[2] Mitchison D, Davies G (2012). The chemotherapy of tuberculosis: past, present and future. Int J Tuberc Lung Dis.

[3] Gillespie SH, Crook AM, McHugh TD, Mendel CM, Meredith SK, Murray SR (2014). Four-month moxifloxacin-based regimens for drug-sensitive tuberculosis. N Engl J Med.

[4] Jindani A, Dore CJ, Mitchison DA (2003). Bactericidal and sterilizing activities of antituberculosis drugs during the first 14 days. Am J Respir Crit Care Med.

[5] Mukamolova GV, Turapov O, Malkin J, Woltmann G, Barer MR (2010). Resuscitation-promoting factors reveal an occult population of tubercle bacilli in sputum. Am J Respir Crit Care Med.

[6] Dhillon J, Fourie PB, Mitchison DA (2014). Persister populations of *Mycobacterium tuberculosis* in sputum that grow in liquid but not on solid culture media. J Antimicrob Chemother.

[7] Dhillon J, Lowrie DB, Mitchison DA (2004). Mycobacterium tuberculosis from chronic murine infections that grows in liquid but not on solid medium. BMC Infect Dis..

[8] Salina EG, Waddell SJ, Hoffmann N, Rosenkrands I, Butcher PD, Kaprelyants AS. Potassium availability triggers *Mycobacterium tuberculosis* transition to, and resuscitation from, non-culturable (dormant) states. Open Biol. 2014;4:140106. http://dx.doi.org/10.1098/rsob.140106 10.1098/rsob.140106 PMC4221891 25320096

[9] Wakamoto Y, Dhar N, Chait R, Schneider K, Signorino-Gelo F, Leibler S (2013). Dynamic persistence of antibiotic-stressed mycobacteria. Science.

[10] Benator D, Bhattacharya M, Bozeman L, Burman W, Cantazaro A, Chaisson R (2002). Rifapentine and isoniazid once a week versus rifampicin and isoniazid twice a week for treatment of drug-susceptible pulmonary tuberculosis in HIV-negative patients: a randomised clinical trial. Lancet.

[11] Hesseling AC, Walzl G, Enarson DA, Carroll NM, Duncan K, Lukey PT (2010). Baseline sputum time to detection predicts month two culture conversion and relapse in non-HIV-infected patients. Int J Tuberc Lung Dis.

[12] Johnson JL, Hadad DJ, Dietze R, Maciel EL, Sewali B, Gitta P (2009). Shortening treatment in adults with noncavitary tuberculosis and 2-month culture conversion. Am J Respir Crit Care Med.

[13] Berry MP, Graham CM, McNab FW, Xu Z, Bloch SA, Oni T (2010). An interferon-inducible neutrophil-driven blood transcriptional signature in human tuberculosis. Nature.

[14] Anderson ST, Kaforou M, Brent AJ, Wright VJ, Banwell CM, Chagaluka G (2014). Diagnosis of childhood tuberculosis and host RNA expression in Africa. N Engl J Med.

[15] Schnappinger D, Ehrt S, Voskuil MI, Liu Y, Mangan JA, Monahan IM (2003). Transcriptional adaptation of *Mycobacterium tuberculosis* within macrophages: insights into the phagosomal environment. J Exp Med.

[16] Tailleux L, Waddell SJ, Pelizzola M, Mortellaro A, Withers M, Tanne A (2008). Probing host pathogen cross-talk by transcriptional profiling of both *Mycobacterium tuberculosis* and infected human dendritic cells and macrophages. PLoS One.

[17] Rohde KH, Veiga DF, Caldwell S, Balazsi G, Russell DG (2012). Linking the transcriptional profiles and the physiological states of *Mycobacterium tuberculosis* during an extended intracellular infection. PLoS Pathog.

[18] Betts JC, Lukey PT, Robb LC, McAdam RA, Duncan K (2002). Evaluation of a nutrient starvation model of *Mycobacterium tuberculosis* persistence by gene and protein expression profiling. Mol Microbiol.

[19] Deb C, Lee CM, Dubey VS, Daniel J, Abomoelak B, Sirakova TD (2009). A novel *in vitro* multiple-stress dormancy model for *Mycobacterium tuberculosis* generates a lipid-loaded, drug-tolerant, dormant pathogen. PLoS One.

[20] Garton NJ, Waddell SJ, Sherratt AL, Lee SM, Smith RJ, Senner C (2008). Cytological and transcript analyses reveal fat and lazy persister-like bacilli in tuberculous sputum. PLoS Med.

[21] Barry CE, Boshoff HI, Dartois V, Dick T, Ehrt S, Flynn J (2009). The spectrum of latent tuberculosis: rethinking the biology and intervention strategies. Nat Rev Microbiol.

[22] Wilson M, DeRisi J, Kristensen HH, Imboden P, Rane S, Brown PO (1999). Exploring drug-induced alterations in gene expression in *Mycobacterium tuberculosis* by microarray hybridization. Proc Natl Acad Sci U S A.

[23] Boshoff HI, Myers TG, Copp BR, McNeil MR, Wilson MA, Barry CE (2004). The transcriptional responses of *Mycobacterium tuberculosis* to inhibitors of metabolism: novel insights into drug mechanisms of action. J Biol Chem.

[24] Makarov V, Manina G, Mikusova K, Mollmann U, Ryabova O, Saint-Joanis B (2009). Benzothiazinones kill *Mycobacterium tuberculosis* by blocking arabinan synthesis. Science.

[25] Karakousis PC, Williams EP, Bishai WR (2008). Altered expression of isoniazid-regulated genes in drug-treated dormant *Mycobacterium tuberculosis*. J Antimicrob Chemother.

[26] Tudo G, Laing K, Mitchison DA, Butcher PD, Waddell SJ (2010). Examining the basis of isoniazid tolerance in nonreplicating *Mycobacterium tuberculosis* using transcriptional profiling. Future Med Chem.

[27] Honeyborne I, McHugh TD, Phillips PP, Bannoo S, Bateson A, Carroll N (2011). Molecular bacterial load assay, a culture-free biomarker for rapid and accurate quantification of sputum *Mycobacterium tuberculosis* bacillary load during treatment. J Clin Microbiol.

[28] Waddell SJ, Laing K, Senner C, Butcher PD (2008). Microarray analysis of defined *Mycobacterium tuberculosis* populations using RNA amplification strategies. BMC Genomics.

[29] Eisen MB, Spellman PT, Brown PO, Botstein D (1998). Cluster analysis and display of genome-wide expression patterns. Proc Natl Acad Sci U S A.

[30] Ernst J, Bar-Joseph Z (2006). STEM: a tool for the analysis of short time series gene expression data. BMC Bioinformatics..

[31] Livak KJ, Schmittgen TD (2001). Analysis of relative gene expression data using real-time quantitative PCR and the 2(-Delta Delta C(T)) method. Methods.

[32] Jolliffe IT (2002). Principal Component Analysis.

[33] Cortes C, Vapnik V (1995). Support-vector networks. Mach Learn.

[34] Meinshausen N, Bühlmann P (2010). Stability selection. J R Stat Soc Ser B (Stat Methodol).

[35] Rondina J, Hahn T, de Oliveira L, Marquand A, Dresler T, Leitner T, Fallgatter A, Shawe-Taylor J, Mourao-Miranda J (2014). SCoRS - a method based on stability for feature selection and mapping in neuroimaging. IEEE Trans Med Imaging.

[36] Tibshirani R (1996). Regression shrinkage and selection via the Lasso. J R Stat Soc Ser B (Stat Methodol).

[37] Cole ST, Brosch R, Parkhill J, Garnier T, Churcher C, Harris D (1998). Deciphering the biology of *Mycobacterium tuberculosis* from the complete genome sequence. Nature.

[38] Shi L, Sohaskey CD, Pfeiffer C, Datta P, Parks M, McFadden J (2010). Carbon flux rerouting during *Mycobacterium tuberculosis* growth arrest. Mol Microbiol.

[39] Rhee KY, de Carvalho LP, Bryk R, Ehrt S, Marrero J, Park SW (2011). Central carbon metabolism in *Mycobacterium tuberculosis*: an unexpected frontier. Trends Microbiol.

[40] Van der Geize R, Yam K, Heuser T, Wilbrink MH, Hara H, Anderton MC (2007). A gene cluster encoding cholesterol catabolism in a soil actinomycete provides insight into *Mycobacterium tuberculosis* survival in macrophages. Proc Natl Acad Sci U S A.

[41] Daniel J, Deb C, Dubey VS, Sirakova TD, Abomoelak B, Morbidoni HR (2004). Induction of a novel class of diacylglycerol acyltransferases and triacylglycerol accumulation in *Mycobacterium tuberculosis* as it goes into a dormancy-like state in culture. J Bacteriol.

[42] Shi L, Sohaskey CD, Kana BD, Dawes S, North RJ, Mizrahi V (2005). Changes in energy metabolism of *Mycobacterium tuberculosis* in mouse lung and under *in vitro* conditions affecting aerobic respiration. Proc Natl Acad Sci U S A.

[43] Kendall SL, Movahedzadeh F, Rison SC, Wernisch L, Parish T, Duncan K (2004). The *Mycobacterium tuberculosis* dosRS two-component system is induced by multiple stresses. Tuberculosis (Edinb).

[44] Gaudion A, Dawson L, Davis E, Smollett K (2013). Characterisation of the *Mycobacterium tuberculosis* alternative sigma factor SigG: its operon and regulon. Tuberculosis (Edinb).

[45] Turapov O, Waddell SJ, Burke B, Glenn S, Sarybaeva AA, Tudo G (2014). Antimicrobial treatment improves mycobacterial survival in nonpermissive growth conditions. Antimicrob Agents Chemother.

[46] Pasca MR, Guglierame P, Arcesi F, Bellinzoni M, De Rossi E, Riccardi G (2004). Rv2686c-Rv2687c-Rv2688c, an ABC fluoroquinolone efflux pump in *Mycobacterium tuberculosis*. Antimicrob Agents Chemother.

[47] Bolla JR, Do SV, Long F, Dai L, Su CC, Lei HT (2012). Structural and functional analysis of the transcriptional regulator Rv3066 of *Mycobacterium tuberculosis*. Nucleic Acids Res.

[48] Walter ND, Dolganov GM, Garcia BJ, Worodria W, Andama A, Musisi E, Ayakaka I, Van TT, Voskuil MI, de Jong BC (2015). Transcriptional adaptation of drug-tolerant Mycobacterium tuberculosis during treatment of human tuberculosis. J Infect Dis.

[49] Prideaux B, Via LE, Zimmerman MD, Eum S, Sarathy J, O'Brien P, Chen C, Kaya F, Weiner DM, Chen PY (2015). The association between sterilizing activity and drug distribution into tuberculosis lesions. Nat Med.

[50] Manina G, Dhar N, McKinney JD (2015). Stress and host immunity amplify *Mycobacterium tuberculosis* phenotypic heterogeneity and induce nongrowing metabolically active forms. Cell Host Microbe.

[51] Sloan DJ, Mwandumba HC, Garton NJ, Khoo SH, Butterworth AE, Allain TJ (2015). Pharmacodynamic modeling of bacillary elimination rates and detection of bacterial lipid bodies in sputum to predict and understand outcomes in treatment of pulmonary tuberculosis. Clin Infect Dis.

