# Supplementary material for: Profiling persistent tubercule bacilli from patient sputa during therapy predicts early drug efficacy
Source: BMC Med. 2016 Apr 7;14:68. doi: 10.1186/s12916-016-0609-3 (PMC4825072; doi:10.1186/s12916-016-0609-3)
Supplement: Additional file 5: — Figure S1. An illustration of the computation process to test associations between transcriptional signatures and patient variables. Figure S2. Venn diagrams describing the overlapping transcriptional signatures of bacilli in sputum relative to aerobic log phase growth. Figure S3. Box and whisker plots mapping the differential expression of gene families. Figure S4. Hierarchical clustering of the mean M.tb transcriptional profiles derived from sputa. Figure S5. Venn diagram highlighting genes significantly induced 3 days after the start of drug therapy. Figure S6. Contrasting patient trajectories as defined by principle component analysis plotting day 0 and day 3 timepoints only. (PDF 526 kb) [file 12916_2016_609_MOESM5_ESM.pdf]

Additional File 5

Figure S1: A diagram illustrating the computation process testing associations between *M.tb* transcriptional signatures and patient clinical/microbiological variables.

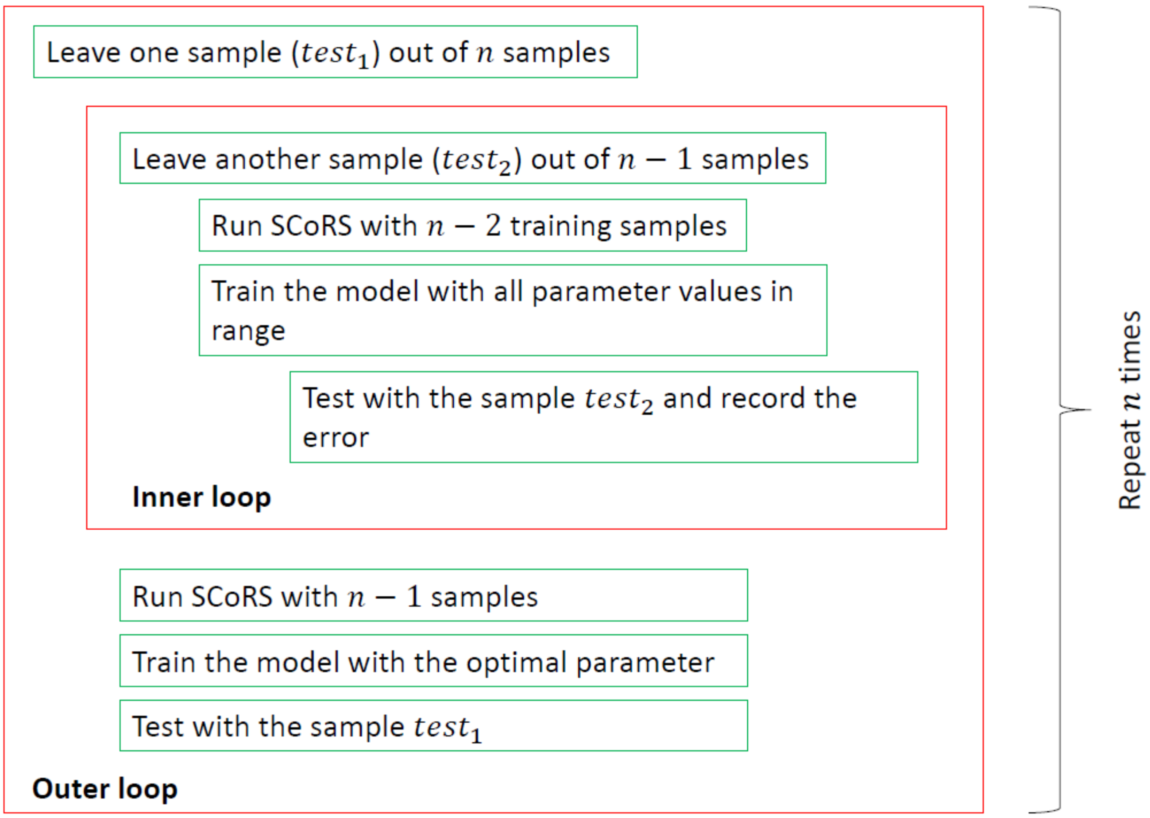

Figure S2: Venn diagrams describing the overlapping transcriptional signatures of bacilli in sputum relative to aerobic log phase growth before chemotherapy (Day 0) and 3 days, 7 days, and 14 days after the start of drug therapy. Genes significantly **(a)** induced or **(b)** repressed at each sputum time interval compared to log phase growth are plotted. These genes are clustered in **Fig. 1** and detailed further in **Table S2**.

a

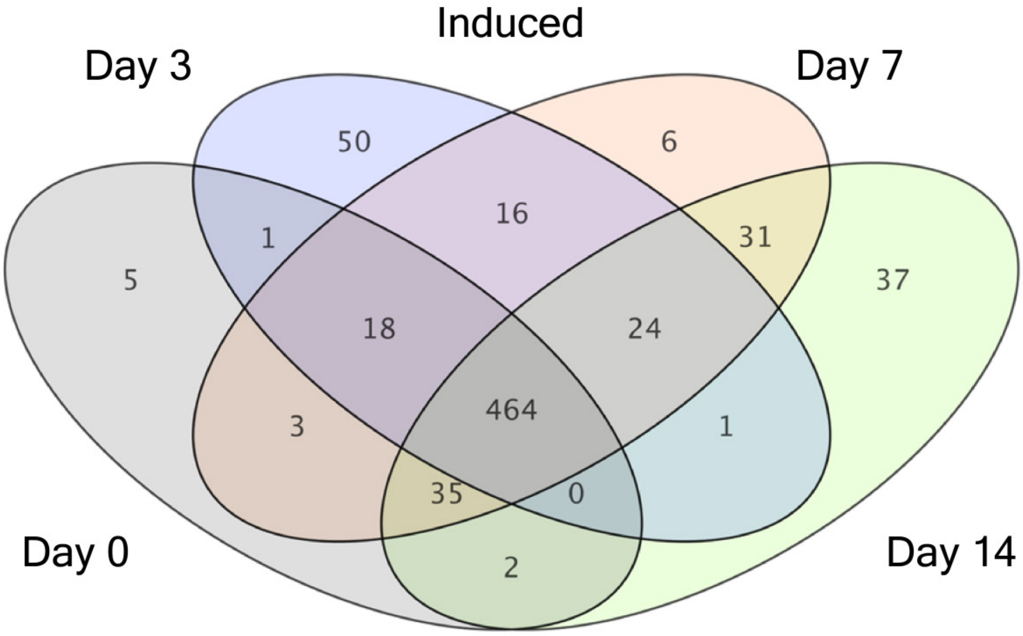

b

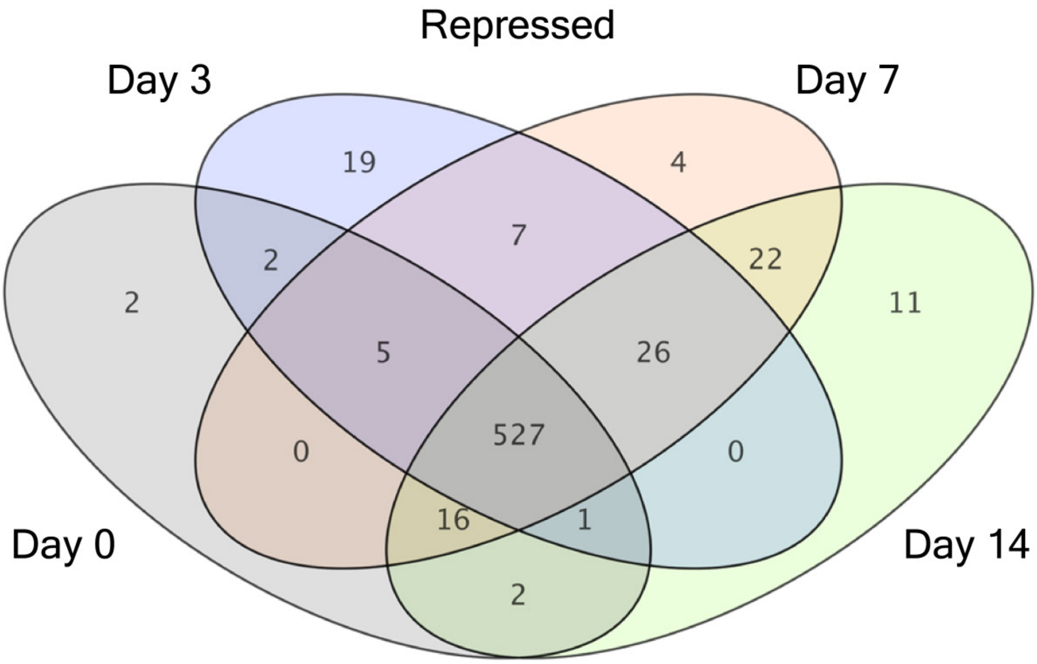

Figure S3: Box and whisker plots mapping the differential expression of genes belonging to ribosome biosynthesis, aerobic respiration and TCA cycle functional categories [1], the DosR-regulon [2], and our previously-defined sputum signature [3] at day 0 (D0) before therapy and 3 days (D3), 7 days (D7), and 14 days (D14) after the start of drug treatment. Expression ratios are plotted in log2 space with the y-axis detailing fold change relative to aerobic log phase growth (marked Log). The *M.tb* sputum transcriptional signature most closely resembled gene expression responses defining reduced growth rate (hg p-values of 4.7×10^{-4} and 6.0×10^{-23} for up- and down-regulated genes respectively [4]); adaptations to the macrophage intracellular environment (hg p-values 2.6×10^{-5} and 1.2×10^{-62} for up- and down-regulated genes respectively [5]); and extracellular *in vivo* growth in a murine hollow-fibre model (hg p-values 4.6×10^{-4} and 1.5×10^{-23} for up- and down-regulated genes respectively [6]). Importantly, the pre-chemotherapy sputum *M.tb* RNA profile closely mirrored that described in an earlier study of expectorated bacilli [3], with hypergeometric p-values of 2.1×10^{-8} and 1.1×10^{-97} for up- and down-regulated genes respectively.

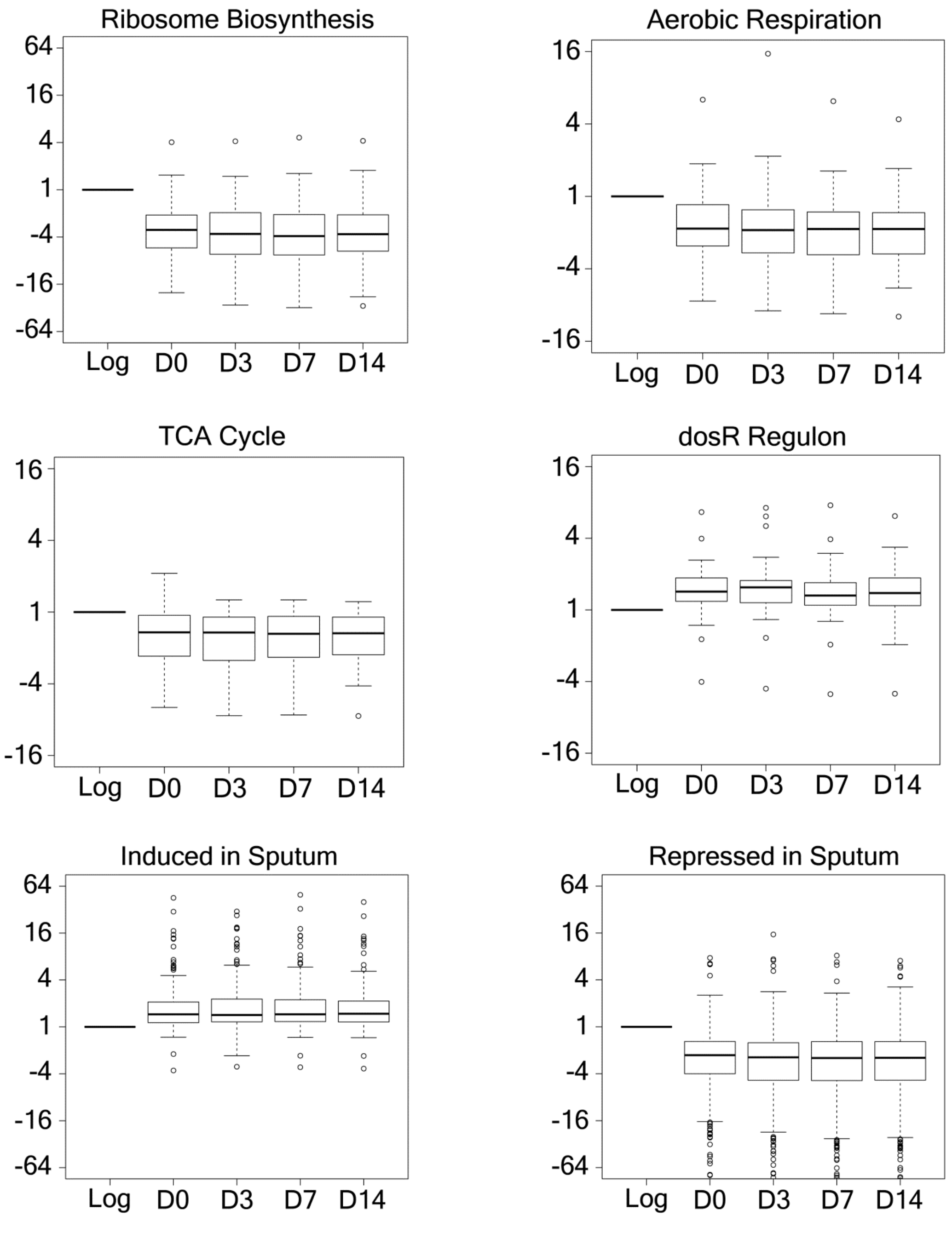

Figure S4: Hierarchical clustering of the mean *M.tb* transcriptional profiles derived from sputa before the start of drug therapy (Day 0) and 3, 7, and 14 days into chemotherapy (Day 3, Day 7, Day 14). The dendrogram is derived from clustering the mean expression profile of each timepoint for all genes.

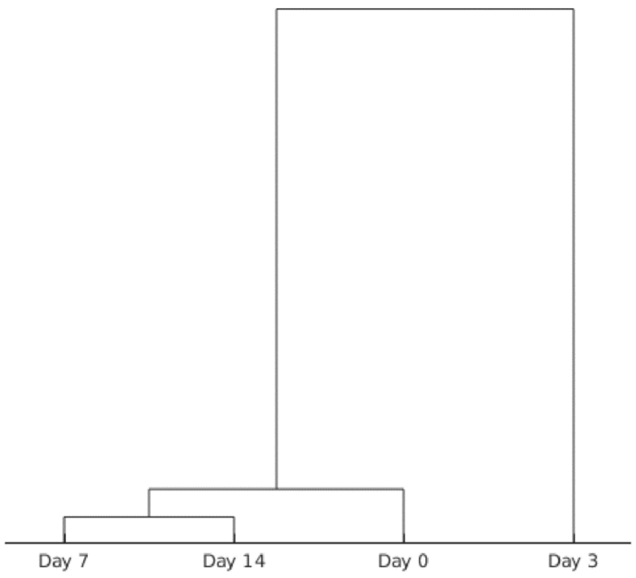

Figure S5: Venn diagram highlighting genes significantly induced 3 days after the start of drug therapy compared to pre-chemotherapy (Day 0), 7 days, and 14 days chemotherapy; identifying conserved differences between the day 3 response relative to other sputa timepoints.

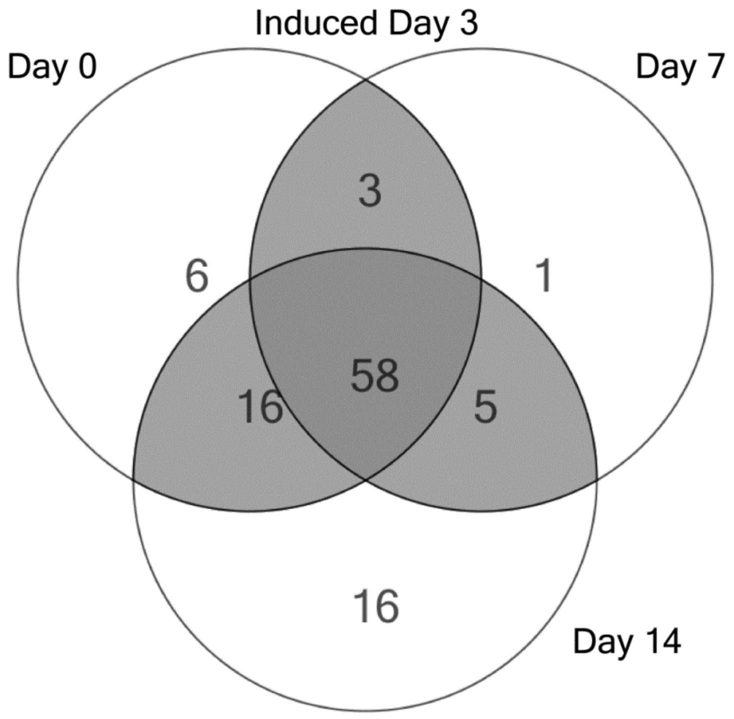

Figure S6: *M.tb* responses to drug therapy result in contrasting patient trajectories as defined by principle component analysis (PCA). The first (PC1) and second (PC2) principle components of the *M.tb* transcriptional signatures from each patient at day 0 and day 3 are plotted in PC space. Each point represents an *M.tb* mRNA profile derived from a patient (colored individually), arrows and dashed lines mark the direction and distance of movement of each patient from day 0 to day 3 highlighting two classes of response South-North or East-West. Patient study identifiers are plotted at day 0; patients with missing day 3 measurements are not plotted.

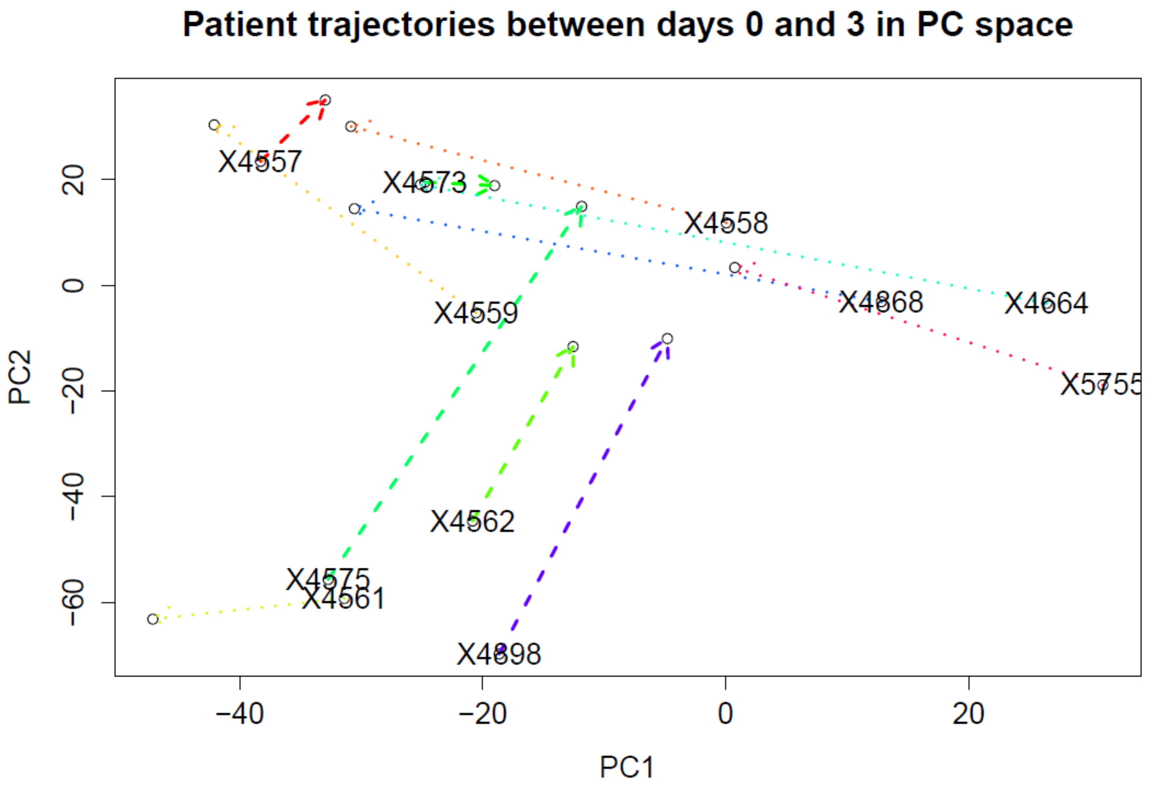

Additional file 1:
Table S1: Participants' molecular, microbiological and clinical parameters.

Additional file 2:
Table S2: *M.tuberculosis* genes significantly differentially expressed in bacilli derived from sputum before the start of chemotherapy (day 0) and 3, 7, and 14 days during treatment compared to log phase aerobic growth. Genes were identified using a moderated t-test (p-value <0.05 with Benjamini and Hochberg multiple testing correction) and fold change >2. These genes are clustered in **Fig. 1a** and mapped in a Venn diagram in **Fig. S2**.

Additional file 3:
Table S3: *M.tuberculosis* genes significantly differentially expressed in sputa over time. Genes were identified using a moderated t-test (p-value <0.05 with Benjamini and Hochberg multiple testing correction) and fold change >1.5. These transcriptional signatures are summarised in **Fig. 2b/c**.

Additional file 4:
Table S4: Quantitative RT-PCR multiplex primer and probe sequences.

References

1. Cole ST, Brosch R, Parkhill J, Garnier T, Churcher C, Harris D, Gordon SV, Eiglmeier K, Gas S, Barry CE, 3rd *et al*: **Deciphering the biology of *Mycobacterium tuberculosis* from the complete genome sequence**. *Nature* 1998, **393**(6685):537-544.
2. Park HD, Guinn KM, Harrell MI, Liao R, Voskuil MI, Tompa M, Schoolnik GK, Sherman DR: **Rv3133c / dosR is a transcription factor that mediates the hypoxic response of *Mycobacterium tuberculosis*** *MolMicrobiol* 2003, **48**(3):833-843.
3. Garton NJ, Waddell SJ, Sherratt AL, Lee SM, Smith RJ, Senner C, Hinds J, Rajakumar K, Adegbola RA, Besra GS *et al*: **Cytological and transcript analyses reveal fat and lazy persister-like bacilli in tuberculous sputum**. *PLoSMed* 2008, **5**(4):e75.
4. Beste DJ, Laing E, Bonde B, Avignone-Rossa C, Bushell ME, McFadden JJ: **Transcriptomic analysis identifies growth rate modulation as a component of the adaptation of mycobacteria to survival inside the macrophage**. *J Bacteriol* 2007, **189**(11):3969-3976.
5. Schnappinger D, Ehrt S, Voskuil MI, Liu Y, Mangan JA, Monahan IM, Dolganov G, Efron B, Butcher PD, Nathan C *et al*: **Transcriptional Adaptation of *Mycobacterium tuberculosis* within Macrophages: Insights into the Phagosomal Environment**. *J Exp Med* 2003, **198**(5):693-704.
6. Karakousis PC, Yoshimatsu T, Lamichhane G, Woolwine SC, Nuermberger EL, Grosset J, Bishai WR: **Dormancy phenotype displayed by extracellular *Mycobacterium tuberculosis* within artificial granulomas in mice**. *JExpMed* 2004, **200**(5):647-657.
